# Dr. Lee on the Function of the Ovaries in Menstruation

**Published:** 1840-10

**Authors:** 


### DR. LEE ON THE FUNCTION OF THE OVARIES IN MENSTRUATION.

In the article on M. Gendrin's Philosophical Treatise of Medicine, in our last Number, p. 75, the following remark was made: "That M. Gendrin should have found the ovaria ruptured in five cases where menstruation was present is a remarkable coincidence. In a communication read at the Medico-Chirurgical Society, by Dr. Robert Lee, last year, a similar occurrence was stated to have been met with by him in two instances. We want but a few more such facts to establish M. Gendrin's hypothesis, or at least to give it the aspect of a law rather than of a mere coincidence; and until these are obtained, we would again repeat —*not proven.*"

In justice to Dr. Lee, we beg to mention that the two instances above alluded to of ruptured Graafian vesicles found in ovaries removed from the bodies of women who had died during menstruation, are merely supplementary to the collection of facts which Dr. L. published seven years ago in the Cyclopædia of Medicine; and, also, that the views he deduced from them are essentially the same as those lately brought forward by M. Gendrin as a new theory of menstruation. We alluded to M. Negrier's claim to priority in opposition to Gendrin; but it is quite evident that neither the one nor the other has any right to be considered *historically* as original observers, however they may have been so in fact. It will be seen from the following extract from the Cyclopædia of Medicine, that Mr. Cruikshank must be looked on as the original observer of the fact, and Dr. Lee the author of additional and confirmatory facts; and indisputably, as far as we can trace, the first propounder of the view that menstruation is connected with certain changes in the ovaria.

"There are certain facts which seem to prove that it is not to the influence of the uterus, but of the ovaria that we are to attribute all the changes which take place in the female pelvis, in the mammæ and uterine system at the period of puberty; and it seems not improbable from the following facts, that it is also to certain changes in the Graafian vesicles at the time of menstruation that all the phenomena of that singular process are to be referred.

"On the 11th of March, 1831, we examined the body of a young woman who died during menstruation from inflammation of the median basilic vein. The left ovarium was larger than the right, and at one point a small circular opening, with thin irregular edges, was observed in the peritoneal coat, which led to a cavity of no great depth in the ovarium. Around the opening, to an extent of three or four lines, the surface of the ovarium was of a bright red colour, and considerably elevated above the surrounding part of the peritoneal coat. On cutting into the ovarium, its substance around the opening and depression was vascular, and several Graafian vesicles of different sizes were observed. The right ovarium was in the ordinary state. Both fallopian tubes were intensely red and swollen, and their cavities were filled with menstrual fluid. The lining membrane of the uterus was coated with the same fluid, and the parietes were soft and vascular. The size of the uterus was not increased.

"In the autumn of the same year a woman under twenty years of age died suddenly from acute inflammation of the lungs while menstruating. The body was examined by Mr. John Prout, and the uterine organs were brought to us for inspection. A red soft elevated portion of the right ovarium was also here observed, and at one part the peritoneal coat to a small extent had been removed. The edges of the opening were extremely thin and irregular, and in the substance of the ovarium under the opening was an enlarged Graafian vesicle filled with transparent fluid. Numerous small blood-vessels were seen running along the peritoneal coat of the ovary to the opening. When the substance of the ovarium was laid open, several vesicles of various sizes, and at different depths, were found imbedded in it. The left ovarium presented a natural appearance. The free extremities of the fallopian tubes were gorged with blood. Their cavities were filled with a red-coloured fluid. The uterus was not enlarged, but the parietes were gorged with blood, and the lining membrane of the fundus was coated with menstrual fluid. A small coagulum of blood likewise adhered to the upper part of the uterus.

"On the 2d of July, 1832, Sir Astley Cooper, to whom the writer had mentioned these cases, sent him the ovarium of a woman who died from cholera while menstruating. The ovarium was much larger than natural, and at one point there was a small irregular aperture in its peritoneal coat through which a portion of a slender coagulum of blood was suspended. On cutting into the substance of the ovarium, it was found to be occupied by three small cavities or cysts, one of which was filled with a clear ropy fluid, another with semi-fluid blood, and the third, which communicated with the opening in the peritoneal coat of the ovarium, with a firm coagulum.

"On the 18th of November, 1832, the uterine organs were removed by Messrs. Girdwood and Webster from the body of a young woman who had died suddenly the preceding day when the catamenia were flowing. Both ovaria were remarkably large, and both fallopian tubes were red and turgid. The peritoneal coat of the left ovarium was perforated at that extremity which was nearest to the uterus by a circular opening, around which aperture for several lines the surface of the ovarium was elevated and of a bright scarlet colour, like extravasated injection The margin of this opening was thin and smooth, and did not appear to have been produced by laceration. Its centre was slightly depressed below the level of the edges, but there was scarcely the appearance of a cavity beneath. The right ovarium was much larger than the left; and, when cut into, a cavity or cyst was found which was filled with half-coagulated blood. The peritoneal coat of this ovarium was entire.

"The uterus was large, and when cut into, the parietes appeared to contain an unusual quantity of blood. The inner membrane was of a bright red colour, and coated with a thin layer of catamenial fluid. Both fallopian tubes were red and turgid, and the interior of the left was filled with menstrual fluid, but nothing in the form of a Graafian vesicle could be detected in the tube. The appearances now described have been accurately represented in a drawing made from the parts within two hours after they came into the author's possession.

"In a paper by Mr. Cruikshank, published in 1797, there is an account of similar appearances having been observed by him in a young woman who had died at the monthly period. 'I have also,' he says, 'in my possession the uterus and ovaria of a young woman who died with the menses upon her. The external membranes of the ovary were burst at one place, from whence I suspect an ovum escaped, descended through the tube to the uterus, and was washed off by the menstrual blood.'" (Cyc. of Pract. Med.—art. Ovaria.)

The following is the case recently communicated to the Medico-Chirurgical Society:

"On the 14th of January, 1837, a woman, thirty-seven years of age, who had long suffered from hysteria, died suddenly in St. George's Hospital during menstruation. No morbid appearance was found to account for her death. A small circular aperture was observed in the peritoneum of the left ovarium, near the point where the corpus fimbriatum is fixed to the extremity of the ovarium. This opening communicated with a cavity in the substance of the ovarium, which was surrounded with a soft yellow substance of an oval shape." (Med. Chir. Transactions, vol. xxii. p. 335.)

